# Mechanism of PVDF Membrane Formation by NIPS Revisited: Effect of Precipitation Bath Nature and Polymer–Solvent Affinity

**DOI:** 10.3390/polym15214307

**Published:** 2023-11-02

**Authors:** Andrey Basko, Tatyana Lebedeva, Mikhail Yurov, Anna Ilyasova, Galina Elyashevich, Viktor Lavrentyev, Denis Kalmykov, Alexey Volkov, Konstantin Pochivalov

**Affiliations:** 1 G.A. Krestov Institute of Solution Chemistry of the Russian Academy of Sciences, 1 ul. Akademicheskaya, 153045 Ivanovo, Russia; avb@isc-ras.ru (A.B.); ltn@isc-ras.ru (T.L.); mihayur@mail.ru (M.Y.); anna.nik.ilyasova@mail.ru (A.I.); denis.kalmykov@ips.ac.ru (D.K.); 2 Institute of Macromolecular Compounds of the Russian Academy of Sciences, 31 Bolshoy pr., 199004 St. Petersburg, Russia; elya@hq.macro.ru (G.E.); lavrentev1949@mail.ru (V.L.); 3 A.V. Topchiev Institute of Petrochemical Synthesis of the Russian Academy of Sciences, 29 Leninsky Prospect, 119991 Moscow, Russia; avolkov@ips.ac.ru

**Keywords:** ultrafiltration membrane, polyvinylidene fluoride, nonsolvent-induced phase separation, ternary phase diagram, mechanism of structure formation

## Abstract

A new interpretation of the mechanism of the polyvinylidene fluoride (PVDF) membrane formation using the nonsolvent-induced phase separation (NIPS) method based on an analysis of the complete experimental phase diagram for the three-component mixture PVDF–dimethyl acetamide (DMAc)–water is proposed. The effects of the precipitation bath’s harshness and thermodynamic affinity of the polymer’s solvent on the morphology, crystalline structure, transport and physical–mechanical properties of the membranes are investigated. These characteristics were studied via scanning electron microscopy, wide-angle X-ray scattering, liquid–liquid porosimetry and standard methods of physico-mechanical analysis. It is established that an increase in DMAc concentration in the precipitation bath results in the growth of mean pore size from ~60 to ~150 nm and an increase in permeance from ~2.8 to ~8 L m^−2^ h^−1^ bar^−1^. It was observed that pore size transformations are accompanied by changes in the tensile strength of membranes from ~9 to ~11 and to 6 MPa, which were explained by the degeneration of finger-like pores and appearance of spherulitic structures in the samples. The addition of water to the dope solution decreased both the transport (mean pore size changed from ~55 to ~25 nm and permeance reduced from ~2.8 to ~0.5 L m^−2^ h^−1^ bar^−1^) and mechanical properties of the membranes (tensile strength decreased from ~9 to ~6 MPa). It is possible to conclude that the best membrane quality may be reached using pure DMAc as a solvent and a precipitation bath containing 10–30% wt. of DMAc, in addition to water.

## 1. Introduction

Membrane technologies continue to attract interest from both scientists and industrial engineers, since they provide the possibility of solving the relevant problems of separation, including the issue of desalinated water deficiency. The possibilities offered by these technologies are due to their cost-efficiency in terms of energy and materials, eco-friendliness and flexibility in operation [1,2,3,4,5,6,7]. Most of the membranes used at present are produced from various polymers, with polyvinylidene fluoride (PVDF) being the main choice for more than a quarter of the global market [8]. Despite the current trend of phasing out the polyfluoroalkyl substances, PVDF remains widely used due to its almost unique combination of characteristics such as chemical stability (especially in relation to acids), high mechanical strength and great thermal stability. Furthermore, polar modifications of PVDF possess piezoelectric and other useful properties [9,10,11]. At the same time, despite its relatively high melting point (178 °C), PVDF is soluble in polar aprotic solvents (dimethyl acetamide (DMAc), dimethyl formamide, N-methyl pyrrolidone, etc.) at room or slightly elevated temperature, which significantly facilitates processing.

At present, the most widespread way of preparing PVDF membranes is the nonsolvent-induced phase separation (NIPS) method [3,12]. This method consists of the preparation of a homogeneous mixture of the polymer and solvent, before it is shaped into the desired form (thin film or hollow fiber) and immersed in a bath with nonsolvent. Mass transfer between the solvent and nonsolvent induces phase separation and/or polymer crystallization and a capillary-porous body spontaneously forms. Pores of this material are filled with an almost pure nonsolvent, which is then removed, usually by drying. Several papers have shown that, depending on the parameters of the NIPS process, membranes with either a symmetric [13,14,15] or asymmetric [15,16,17] structure can be prepared. Data on mean through-pore sizes indicate that the PVDF membranes prepared via NIPS can be used in nano- [18,19] ultra- [20,21,22,23,24] and microfiltration [24,25] processes. In the cited papers, the membrane performance was assessed using water solutions of bovine serum albumine [18,19,20,21,22], several dyes [18,19,20], polymers [23], humic acid [20,22,25] and inorganic salts [19].

At the same time, despite the fact that the NIPS method has been successfully used for the preparation of porous materials for more than 50 years [26], the question of the structure formation mechanism during NIPS remains controversial [27,28,29,30,31,32,33,34]. Moreover, the number of publications devoted to this problem has increased in recent years [35,36,37,38,39,40,41,42,43]. For example, to describe the mechanism of the so-called finger-like pores’ (FLPs) formation, several approaches, such as the coalescence of the smaller droplets [44,45,46] and the morphological instability of the interface between two liquids (viscous fingering) [37,47,48,49], were proposed. In our recent papers [50,51,52], we assumed that the reason for the initiation and growth of FLP is the nonuniformity of the rate of nonsolvent diffusion through the matrix and open pores in the skin layer.

As an analysis of the science literature shows, there are several ways to control the structure and properties of the membranes prepared via NIPS. For example, they can be controlled by changing the composition of the dope solution (polymer concentration [53,54], presence and concentration of inorganic [55,56] and polymeric [55,57] additives), or the thermodynamic affinity of the solvent to the polymer, which, in turn, depends on the solvent’s chemical nature [58,59,60] or can be changed by adding a nonsolvent to the dope solution [61,62], the temperature of the process [63,64], and the nature (harshness) of the precipitation bath.

The harshness of the precipitation bath in this case is a property reflecting the precipitation ability of polymer, its ability to extract solvent from binary polymer–solvent mixtures, etc. The effect of precipitation bath harshness on the morphology and properties of the membranes was studied in several papers; for example, [64,65,66,67,68]. In [67], it is shown that the partial replacement of water with methanol, ethanol and isopropanol in precipitation baths leads to a decrease in the porosity and mechanical properties of the obtained PVDF hollow fiber membranes. In [68], it is shown that, despite the quite pronounced effect of the replacement of water with ethanol in a precipitation bath on the morphology and water contact angle of the membranes (from ~84° for pure water in precipitation bath to 116° for 50% wt. ethanol and 150° for 96% wt. ethanol), the transport properties were almost identical for membranes prepared by immersion in 50, 75 and 96% wt. ethanol (for example, distilled water permeance in vacuum membrane distillation test at 50 °C was in the range 44–52 L m^−2^ h^−1^). It should be noted that, commonly, the harshness of the precipitation bath for PVDF membrane preparation is controlled by the full or partial replacement of water with different alcohols.

The effect of the thermodynamic affinity of the solvent for a polymer (the nature of the solvent) on the structure and properties of polymeric membranes prepared via NIPS was studied in [57,58,59]. It was shown that the nature of the solvent that is used may affect the mechanical and transport properties of the membranes. In [69,70], it is also noted that, depending on the solvent that is used, PVDF may crystallize into different polymorphs.

To describe the membrane formation process in terms of thermodynamics, one would need phase diagrams for each of the ternary (quasiternary) systems that were used.

At the same time, it is well known that the harshness of the precipitation bath may be controlled by the addition of a solvent to the nonsolvent [71], and the thermodynamic affinity between the solvent and the polymer may be changed by the addition of nonsolvent to the dope solution [61,62]. In these papers, it was proposed to use the so-called “incipient dope” as a membrane-casting solution. This “incipient dope” is a ternary mixture of polymer, solvent and nonsolvent, which is in a state of nonequilibrium from the thermodynamic perspective but remains in a metastable homogeneous state just before the membrane formation process. In [72], it was shown that the use of “incipient dope” instead of a binary polymer–solvent mixture leads to an increase in the membrane permeance and the mean pore size, despite the decrease in overall porosity. However, different concentrations of nonsolvent in the dope solution were not discussed in the cited papers.

If the nonsolvent harshness and polymer–solvent affinity are regulated in this way, to describe the thermodynamics of the membrane formation process, only one phase diagram for the chosen ternary system (semicrystalline (SC) polymer–solvent–nonsolvent) would be sufficient.

Recently, we [52] plotted, for the first time, a complete experimental phase diagram for tmembrane preparation via the widely used NIPS mixture of PVDF, DMAc and water. The plotted diagram and thermal behavior data from this paper were used for a discussion of the results obtained in this work.

The purpose of the present study was to systematically investigate the effects of the precipitation bath’s harshness and polymer–solvent affinity on the morphology, structure formation mechanism, mechanical, transport and other properties of the membranes prepared via NIPS. The harshness of the precipitation bath was adjusted by the addition of DMAc to the water, and polymer–solvent affinity was changed by the addition of water to the DMAc. As far as we are aware, this is the first attempt to investigate the effects of these parameters on membrane morphology and properties from a purely thermodynamic perspective using a single ternary phase diagram.

## 2. Materials and Methods

### 2.1. Materials

In the present work, PVDF (Solvay Solef 6020, Solvay S.A., Brussels, Belgium) with a melting temperature *T*_m_ of 175.8 °C, melt flow index of 4.5 g/10 min (21.6 kg, 230 °C), crystallinity degree α of 73.5% and density ρ of 1.778 g cm^−3^ was used. DMAc (“Component-reactive”, Ltd., Moscow, Russia) with a refractive index of 1.435 and density of 0.940 g cm^−3^ was used as a solvent. The listed characteristics of the polymer and the solvent were determined in our previous paper [52]. Distilled water was used as a nonsolvent.

### 2.2. Methods

#### 2.2.1. Membrane Preparation

The membrane samples were prepared as follows. A total of 15% wt. of PVDF powder and 85% wt. of either pure DMAc or a previously prepared mixture of DMAc with water containing 2, 4 or 6% wt. of the latter were placed in the vial and heated to 70 °C. The mixtures were thermostated and occasionally shaken for a time sufficient to form a homogeneous mixture. The obtained mixtures were cooled down to room temperature, and a thin film (membrane precursor) on a polyethylene terephthalate substrate was formed using a casting knife (knife height 450 μm, substrate thickness 50 μm). Then, the film, together with the substrate, was transferred to a precipitation bath (bath modulus of ~1:250) composed of either pure water or its mixture with DMAc (10, 30, 50, 70% wt.) for 16 h. After that, the samples without substrate were transferred to another bath filled with pure water for 24 h in order to remove traces of DMAc. Then, the membrane samples were removed from the bath and dried at room temperature to a constant mass. Compositions of the dope solutions and precipitation baths used to prepare the membranes are given in Table 1.

#### 2.2.2. Evaluation of Membrane Shrinkage and Water Contact Angle

Shrinkage of the membranes (*Sh*) was determined by the measurement of their linear dimensions (length and width) before (*l*_0_) and after drying (*l*_f_), and calculated using the following equation:

(1) $$Sh=\left(\frac{l_{0}-l_{f}}{l_{0}}\right)*100\%,$$

Shrinkage values along the length and width of membranes were almost identical. Mean values of shrinkage were calculated from five measurements.

Water contact angle was estimated via digital analysis of the photographs for 5 μL droplets of distilled water placed on the surface of the membrane. The mean value of water contact angle was calculated from five measurements at different areas of the membranes.

#### 2.2.3. Mechanical Properties

Tensile strength (σ) and relative elongation at break (ε) of the membranes were measured using an I–11M Instrument (“Tochpribor” Ltd., Ivanovo, Russia). Membrane samples were cut into rectangles of 25 mm in length and 3 mm in width and clamped in the instrument so that length of the unclamped part was 15 mm. Extension rate was 50 mm min^−1^. Mean value of the tensile strength was calculated from five measurements according to Equation (2):

(2) $$\sigma =\frac{F_{max}}{S_{0}},$$

where *F_max_* is the breaking force and *S*_0_ is the area of a cross-section of the sample. Elongation at break was calculated according to Equation (3):

(3) $$\varepsilon =\left(\frac{l_{br}}{l_{0}}\right)*100\%,$$

where *l_0_* and *l_br_* are lengths of the unclamped sample before the experiment and at break, respectively.

#### 2.2.4. Membrane Morphology

Morphology of the membrane surfaces and cross-sections was investigated using the Quattro S (Thermo Fisher Scientific, Brno-Černovice, Czech Republic) scanning electron microscope. The images were constructed from secondary electrons using a lower detector. Accelerating voltage was 5 kV. To prepare the cross-sections, the membranes were broken in liquid nitrogen medium. The samples were coated with gold using a sputter-coater, Quorum Q150es plus (Quorum Technologies, Ltd., Laughton, UK), before the study.

#### 2.2.5. Transport Properties of the Membranes

Mean through-pore size and permeance were determined using Poroliq 1000 ML porometer (Porometer Ltd., Nazareth, Belgium). The operating principle of this instrument is based on the displacement of the wetting liquid with a nonwetting one. Details of the experimental technique are described elsewhere [73]. The wetting and nonwetting liquids were saturated by the solutions of water in isobutanol and isobutanol in water, respectively. Permeance values were assessed using the latter.

#### 2.2.6. X-ray Investigations of the Membrane Samples

Wide-angle X-ray scattering studies were performed using a DRON 2.0 diffractometer (Burevestnik SPE, Saint Petersburg, Russia) with CuKα radiation. The recording was carried out in transmission mode. The degree of crystallinity and content of crystalline modifications in the membrane samples were determined.

## 3. Results and Discussion

### 3.1. Themodynamic Discourse of the Membranes Structure Formation Process via NIPS

**Figure 1:**
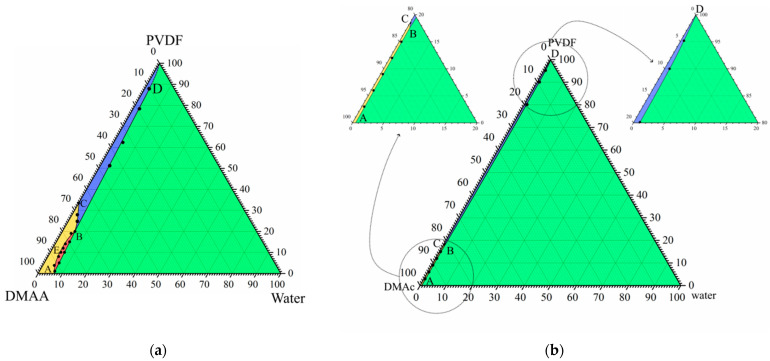
(**a**) Ternary phase diagram for the PVDF–DMAc–water at 60 °C. Reprinted with permission from [52]. (**b**) Ternary phase diagram for the same mixture at 25 °C plotted using data from the cited paper.

Figure 1a shows the phase diagram for the PVDF–DMAc–water ternary mixture at 60 °C, which we were able to plot in our previous work [52]. In this phase diagram, the AEB curve is the liquid equilibrium binodal. The ABC curve is a boundary-separating domain where PVDF is completely amorphous (yellow and red) and in a partially crystalline (blue and green) state in the three-component mixtures. The SC polymer is able to swell in a mixture of solvent (DMAc) and nonsolvent (water), which can be considered a cosolvent. Thus, the BD curve reflects the dependence of SC PVDF swelling degree on the ratio between DMAc and water.

The mentioned curves delineate four domains in the phase diagram. Single-phase homogeneous molecular mixtures of all three components exist in the yellow domain. The red region is the two-phase domain where two liquid solutions with different polymer concentrations coexist. The somposition of the polymer solution in the cosolvent is determined by AE fragments and the composition of the cosolvent solution in the amorphous polymer is determined by EB fragments. Swelling in the cosolvent SC polymer (which can be considered a metastable gel with crystallites acting as physical crosslinks) exists in the blue domain. Such gels coexist with the liquid mixture of solvent and nonsolvent in the green domain, while their composition is determined by the BD curve.

The topology of any ternary phase diagram is dependent on temperature. We used the data reported in [52] to plot the phase diagram for this mixture at 25 °C (Figure 1b). The position of the ABC curve was obtained by graphical interpolation and points on the BD curve were determined as compositions of the mixture in the intersection points of isotherm at 25 °C, with curves in the temperature–composition phase diagrams for the quasibinary mixtures of PVDF and cosolvents of various compositions. One can see that this quasi-equilibrium phase diagram at 25 °C does not contain liquid equilibrium binodal, and the yellow domain (where mixtures are homogeneous) is significantly smaller than that in the phase diagram at 60 °C.

If the NIPS process reached equilibrium, and was thus completely governed by thermodynamics, from the phase diagram shown in Figure 1b, it would be expected that the immersion of a homogeneous polymer–solvent mixture into the nonsolvent bath would result in polymer crystallization. In the real experiment, however, since the polymer crystallization process is sluggish, in similar systems, liquid–liquid phase separation almost always occurs prior to crystallization [31,70,74,75,76]. Therefore, despite the fact that the phase diagram shown in Figure 1b correctly describes the thermodynamic behavior of the system, in nonequilibrium conditions of real experiments, the ABC curve shifts to the right, and the topology of the “dynamic” phase diagram becomes similar to that shown in Figure 1a. It should be noted that the experimental construction of the exact “dynamic” ternary phase diagram is impossible.

General principles of the structure formation process, governed by the “dynamic” phase diagram, similar to the one shown in Figure 1a, can be described as follows [50,51,52]. First, the solvent is rapidly extracted from the surface of the liquid film immersed in the nonsolvent due to the high affinity between the solvent and the nonsolvent. As a result, the polymer concentration in the surface layer increases significantly. Thus, the formed layer starts to act as a barrier to further mass transfer between the solvent in the bulk of membrane precursor and nonsolvent in the precipitation bath. Nonetheless, it contains some through pores. Obviously, the mass transfer rate through the open pores formed in the surface layer and through its “monolithic” parts must be different. Therefore, under the through pores in the surface layer, the FLP begins to grow, while under the nonporous parts of the surface layer, the front of liquid–liquid microphase separation moves, with a little delay, into the bulk of the membrane precursor. With an even longer delay, the front of polymer crystallization also moves from the surface layer into the bulk of the membrane precursor, and the formed microporous structure becomes fixed. Simultaneously with crystallization, microphase separation also occurs in amorphous regions of the polymer-rich phas. This process, also known as the microphase separation of a gel, leads to the formation of small droplets of the polymer-lean liquid inside the amorphous regions of the SC polymer. Pores with a size of less than 100 nm remain after the removal of this liquid. 

The details and peculiarities of the described mechanism of structure formation will change with variations in the polymer–solvent thermodynamic affinity and harshness of the precipitation bath. To elucidate these changes, we studied the membranes prepared from mixtures summarized in Table 1.

### 3.2. Effect of Precipitation Bath Harshness on Morphology and Properties of the Membranes

To study the effect of precipitation bath composition, the nonsolvent (water) was partially replaced by the solvent (DMAc). It should be noted that using this method to control the harshness of the precipitation bath is easier in terms of technology than the common practice of changing the chemical nature of the nonsolvent (for example, the replacement of water with alcohol) since it does not require complex separation processes for the three-component mixtures (water + alcohol + solvent).

#### 3.2.1. Effect of Precipitation Bath Harshness on the Structure Formation Process and Morphology of the Membranes

It is well known that DMAc and water are able to mix in any ratio at room temperature. At the same time, water is practically incompatible with PVDF. Thus, it may be claimed that, regardless of the ratio of DMAc and water in the precipitation bath, only two mass transfer processes are realized between the membrane precursor and bath during the NIPS process. Specifically, DMAc is extracted from the membrane precursor into the precipitation bath and water diffuses into the membrane precursor. An increase in DMAc concentration in the precipitation bath results in a decrease in the thermodynamic driving force (and, consequently, rate) of these mass transfer processes.

**Figure 2:**
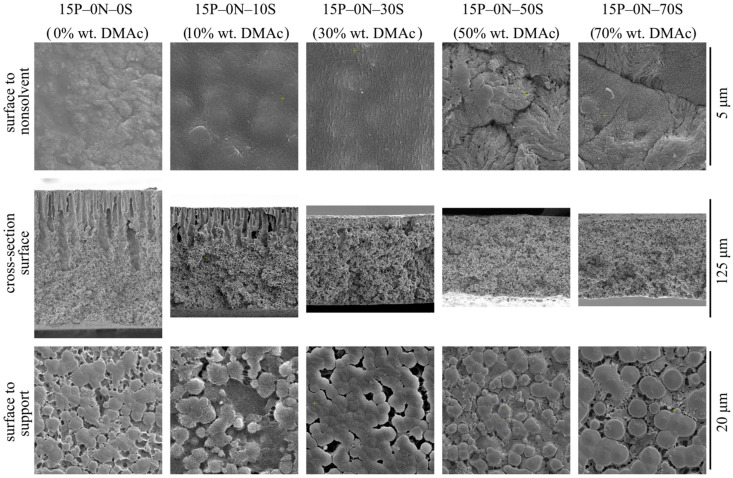
SEM images of the surface that was in contact with the nonsolvent (**upper row**), cross-section (**middle row**), and the surface that was in contact with the PET substrate (**bottom row**) of the membrane samples prepared using precipitation baths of different compositions. Sample code and mass fraction of DMAc in the precipitation bath are shown above the images.

Figure 2 shows SEM images of the surface that was in contact with the precipitant (upper row), cross-section surface (middle row), and surface that was in contact with PET substrate (bottom row) of the membrane samples 15P–0N–0S, 15P–0N–10S, 15P–0N–30S, 15P–0N–50S, and 15P–0N–70S.

From the images presented in the upper row of Figure 2, one can see that the surface porosity increases with an increase in the solvent concentration in the precipitation bath but remains quite low for all samples. In other words, the obtained membranes contain a skin layer regardless of the precipitation bath composition. The transport properties of the membranes and selectivity are limited by the characteristic pore size in this layer. SEM data provide some information on pore size in the skin layer, and it can be argued that pore size changes from being almost indiscernible at a resolution that is possible for the SEM (10–20 nm) to being clearly visible in the images (40–60 nm). However, porosimetry methods are able to provide more precise data on pore size (see Section 3.2.2).

The observed tendencies can be explained by considering the structure formation mechanism of the surface layer. It is accepted that a low-porous selective surface layer forms during NIPS, as follows. After immersion of the polymer–solvent mixture into the nonsolvent, the solvent is extracted from the surface layer into the precipitation bath at a very high rate. Thus, the polymer concentration in this layer is greatly increased and after phase separation in this layer, the volume fraction of pores (containing polymer-lean liquid) is greatly reduced. Furthermore, the higher the solvent concentration in the precipitation bath, the weaker the extraction ability of this mixed precipitant. Therefore, it is to be expected that porosity and pore size in the skin layer of the membranes increase with an increase in DMAc concentration in the precipitation bath.

In addition to the observed trends, Figure 2 shows that the surface of the 15P–0N–50S and 15P–0N–70S samples is more rough (wrinkled) and consists of clearly visible spherulitic structures. This peculiarity of the structure is also a result of the decrease in the solvent extraction rate that occurs with an increase in DMAc concentration in the precipitation bath. In the samples obtained via immersion into harsh baths, the surface layer was rapidly enriched by the polymer, and thus quickly crystallized from many nuclei, forming a large number of very small spherulites. In contrast, in the samples obtained via immersion into soft baths, the extraction rate was lower, so the polymer concentration and crystallization rate was lower; therefore, bigger and more pronounced spherulites were able to grow.

SEM images of the membranes’ cross-section are shown in the middle row of Figure 2. An analysis of these images, taking into account the ideas formulated by us earlier [50,51,52], allows for the following conclusions.

An increase in solvent concentration in the precipitation bath leads to a decrease in the obtained membrane thickness. This can be explained as follows. Immersion of the membrane precursor into the precipitation bath leads, at first, to the formation of a surface layer that starts to act as a barrier to the further diffusion of the solvent into the bath and nonsolvent into the precursor. It was shown that an increase in solvent concentration in the precipitation bath results in an increase in the porosity (and thus permeance) of this layer. In other words, its barrier properties become less pronounced and the rate of diffusion processes through this layer should increase. On the other hand, an increase in solvent content in the precipitation bath decreases the driving force of these diffusion processes. It can be assumed that these two factors compensate each other. At the same time, it is known [51] that an increase in solvent concentration in the precipitation bath leads to a decrease in the rate of the structure formation front movement into the bulk of the membrane precursor. The slower the movement rate of the structure formation front, the more time is available for extraction of the solvent from the homogeneous part of the membrane precursor (layers located deeper under the structure formation front). Therefore, the polymer concentration in these layers increases with an increase in DMAc concentration in the precipitation bath. Obviously, after realization of phase separation in condictions that are more enriched by the polymer layers, less porous structures are produced, and so the overall porosity (volume and thickness) decreases.

FLPs are only present in samples 15P–0N–0S, 15P–0N–10S, and 15P–0N–30S. Moreover, while in the first two samples, FLPs are initiated right under the layer of skin, in the 15P–0N–30S sample, FLPs are much less pronounced and first appear at a distance of ~10 μm from the surface. This transformation of the structure can be explained by adopting the assumption proposed in [50,51,52] that the reason for FLP formation is the inequality of the nonsolvent supply through different regions of the surface layer. 

In harsh baths, the structure of the surface layer forms fast and FLP nuclei appear right under the through pores in the surface layer (right under the regions where the nonsolvent supply is at the highest rate). As the structure formation process continues, more and more open pores are formed in the surface layer, the nonsolvent supply rate through different regions of the surface layer equalizes, and the growth of DLP terminates. 

In the precipitation baths with moderate harshness, the structure formation in the surface layer is slower. It can be assumed that, at the first stages of structure formation, the nonsolvent supply rate through the surface layer was uniform. At this stage, a sponge-like layer with a thickness of ca. 10 μm formed. At the same time, structure formation process in the surface layer continued and some regions (where, due to fluctuations in concentration, more open pores appeared) became more permeable than others. Thus, FLPs started to grow in these regions. Then, as in the above case, the formation of even more pores in the surface layer resulted in equalization of the nonsolvent supply rate and FLPs stopped their growth. 

At last, in soft baths, the formed surface layer limits the diffusion properties very little. In this case, the nonsolvent supply rate in different regions is uniform throughout the structure formation process. If the assumption that the reason for FLP growth is an unequal nonsolvent supply, this would explain why no FLPs are observed in samples 15P–0N–50S and 15P–0N–70S.

Although, at first glance at the images in the bottom row of Figure 2, it seems that the structures are different, all these structures are composed of the partly impinged spherulites with a diameter of ~1–5 μm. The porosity of the bottom surface of the membranes is similar for all samples. In other words, the harshness of the precipitation bath does not affect the morphology of the membrane surface that was in contact with the PET substrate. 

**Figure 3:**
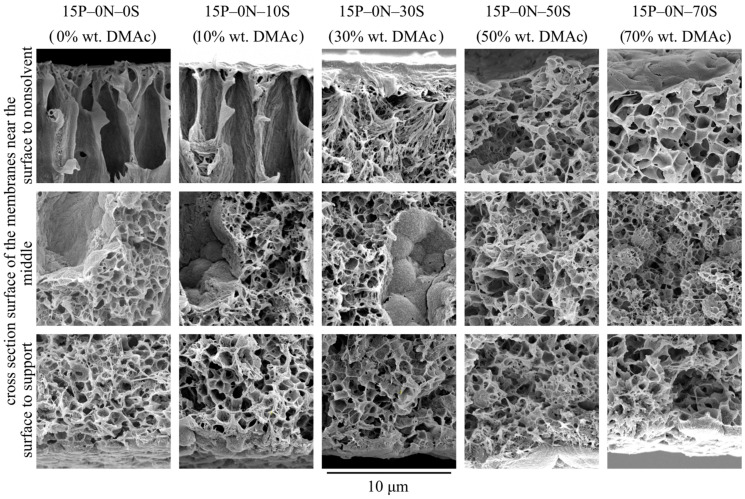
SEM images of the cross-section near the surface that was in contact with precipitation bath (**upper row**), near the middle of the sample (**middle row**) and near the surface that was in contact with PET substrate (**bottom row**) of the membranes prepared using a precipitation bath of different compositions. Sample code and mass fraction of DMAc in the precipitation bath are shown above the images.

Figure 3 shows SEM images of the membrane sample cross-section that was obtained at a higher magnification, near the membrane surfaces and their middle. From an analysis of the images, the following may be concluded.

1. The morphology of the 15P–0N–0S and 15P–0N–10S samples is very similar. Nonetheless, even small transformations in the morphology lead to noticeable changes in the membrane’s mechanical and transport properties, as will be shown below.
2. The porosity (except for the FLP) of the samples in the upper part of the membrane (that was in contact with the nonsolvent) increases with an increase in DMAc concentration in the bath. This is due to the abovementioned decrease in the extraction ability of the precipitation bath.
3. In the walls of the upper part of FLP (Figure 3, samples 15P–0N–0S and 15P–0N–10S), there are dendritic lamellar structures containing small pores in the interlamellar spaces. This fact agrees well with the discourse on structure formation mechanism outlined in Section 3.1. Specifically, the microphase separation of gel that occurs during NIPS simultaneously with the polymer crystallization leads to the formation of small droplets of the polymer-lean phase in the amorphous interlamellar regions.
4. The walls of the lower part of the FLP in samples 15P–0N–0S and 15P–0N–10S (and for the 15P–0N–30S sample all the walls of the FLP) are composed of porous spherulites (later in the present paper; type 1 spherulites). A cross-section of these spherulites shows a typical sponge-like structure. This means that crystallization of the polymer in these samples occurred in the mixture that had already undergone liquid—liquid phase separation with the formation of polymer-lean droplets emulsion in the polymer-rich phase. The spherulites growing in the polymer-rich dispersion medium “encircled” the droplets of the polymer-lean liquid. The sponge-like structure of the membranes is composed of such spherulites. In SEM images, however, it is most noticeable in areas where the growth of spherulites stopped abruptly (i.e., on the surface of FLP and on the surface in contact with the PET substrate).
5. The structure of the 15P–0N–50S and 15P–0N–70S does not contain FLP. However, in these samples, as well as in sample 15P–0N–30S, one can notice spherical particles (later in the present paper; type 2 spherulites) decorated with the “web” of the sponge-like structure.

**Figure 4:**
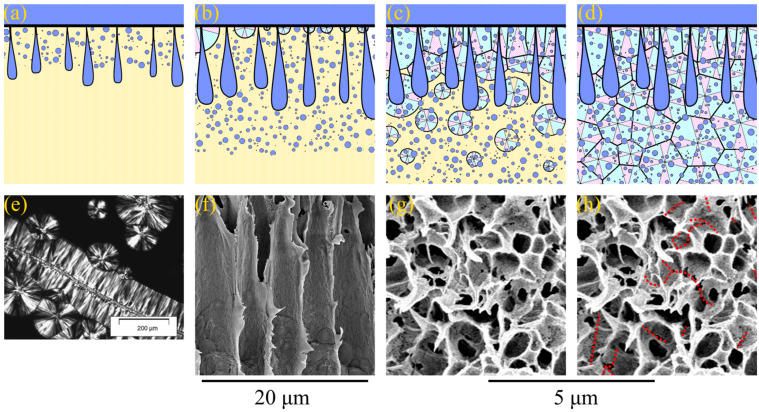
(**a**–**d**) Scheme illustrating the mechanism of formation of transcrystalline layer, FLP and sponge-like structure in the membranes formed via NIPS. See the explanation in the text. (**e**) The photo and example of the transrystalline layer in the SC polymer sample, taken using a polarized light optical microscopy and reprinted with permission from [80], and (**f**) SEM image of the cross-section of the membrane sample 15P–0N–0S illustrating the transcrystalline structure in the upper parts of the FLP walls. (**g**) High-magnification SEM image of the sponge-like structure in the 15P–0N–0S sample. (**h**) The same image, with red dotted lines indicating the boundaries where spherulites impinged on each other.

**Figure 5:**
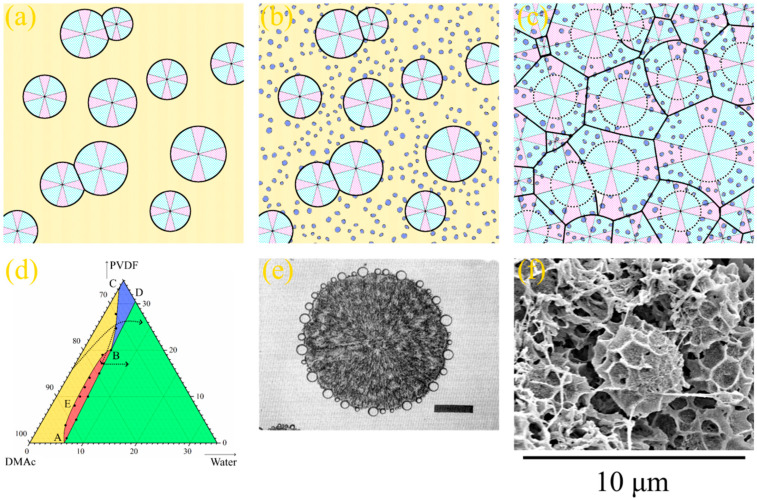
(**a**–**c**) Scheme illustrating the mechanism of the type 2 spherulites’ formation. See the explanation in the text. (**d**) Fragment of the PVDF–DMAc–water ternary phase diagram with the arrows indicating the “composition paths” that ensure the formation of the type 2 spherulites. (**e**) Photograph obtained using a polarized light optical microscope of a poly-ε-caproplactone spherulite, on the surface of which droplets of the polystyrene-rich liquid nucleate; scale bar is 100 μm; reprinted with permission from [83]. (**f**) SEM image of the cross-section of the 15P–0N–70S sample near the surface that was in contact with PET substrate, where the type 2 spherulites enclosed by a sponge-like structure are clearly visible.

The mechanism of the type 2 spherulites’ formation is essentially different for the mechanism of structure formation of type 1 spherulites. Let us discuss these mechanisms using schemes shown in Figure 4 and Figure 5.

In the first case, right after the formation of the skin layer, the realization of the mass transfer processes results in liquid–liquid phase separation with the formation of both FLP and a sponge-like structure in the matrix between them (Figure 4a). After some time, the front of microphase separation catches up and even overtakes the FLP growth front; therefore, FLP stops growing (Figure 4b). At the same time, with the advancement of phase separation fronts into the bulk of the membrane precursor, the polymer starts to crystallize in the polymer-rich phase form, with the surface in contact with the precipitation bath (Figure 4b). Since all the crystallization nuclei are located in the surface layer, the spherulites predominantly grow in the direction normal to the interface between the membrane precursor and nonsolvent. This directional growth in spherulites is responsible for the observed dendritic structures in the upper part of the FLP walls. It should be noted that a similar structure composed of spherulites that grow in the same direction is the subject of a large number of studies [77,78,79,80]. In these papers, the process of polymer crystallization in composites, with fibers acting as nucleators, was investigated, and the formed structure was called transcrystalline. Figure 4e shows a polarized light optical micrograph of an example of such a structure, adopted from [80]. Figure 4f shows a fragment of an SEM image of the 15P–0N–0S sample, where the dendritic structure of the transcrystalline layer in the upper part of the FLP walls is clearly visible. In the bottom part of FLP, the transcrystalline structure transforms into a regular spherulitic one. This is because, after some time, spherulites start to nucleate in random points of the membrane precursor bulk. From this point, spherultes grow omnidirectionally, and the transcrystalline structure gradually transforms into common spherulitic one (Figure 4c). The spherulites growing in the polymer-rich phase “envelop” [81,82] the droplets of the polymer-lean phase and impinge on each other across all the edges (Figure 4d).

A detailed analysis of Figure 3 reveals that the sponge-like structure contains cracks in the matrix located in the regions where different spherulites are impinged during crystallization. Figure 4g shows a high-magnification SEM image of the sponge-like structure in the 15P–0N–0S sample, and Figure 4h shows the same image with red dotted lines indicating the discussed cracks. If the growing spherulite faces an obstacle (FLP or substrate), its growth terminates abruptly and the structure of such spherulites is clearly visible in the SEM images. It should be noted that the sizes of FLP, spherulites and pores in the sponge-like structure are changed in the scheme shown in Figure 4 and Figure 5 for better visual perception. As in the Figure 3 and Figure 4f–h, in real samples, the diameter of FLP reaches several tens of micrometers, which is significantly bigger than the size of the spherulites, which have a diameter of several micrometers and are several times bigger than the mean pore size in the sponge-like structure. These changes, however, do not change the majority of the above discussion.

As mentioned above, the type 2 spherulites are formed only at some distance from the surface layer in samples 15P–0N–30S, 15P–0N–50S, and 15P–0N–70S (see Figure 3). In the vicinity of the surface layer, these samples exhibit a sponge-like structure, which is formed by the mechanism described in Figure 4. The only difference is that FLPs did not appear in these samples.

Figure 5 illustrates the process of structure formation at some distance from the surface layer where type 2 spherulites were detected. Since the process of structure formation is not achieved in equilibrium and the crystallization process is slow, PVDF does not start to crystallize immediately after crossing the AB line in Figure 1b but after a certain degree of supersaturation by water. At the same time, since an increase in DMAc content in the precipitation bath leads to the deceleration of the mass transfer processes, the supersaturation degree that is sufficient for the initiation of crystallization process also decreases. As a result, the layers a certain distance from the surface layer first cross the BC curve on the phase diagram (as shown schematically by the arrow for PVDF–DMAc axis on the Figure 5d). Crossing this line leads to spontaneous nucleation and the growth of PVDF spherulites (Figure 5a). As the crystallization process continues, the polymer chains are “pulled” to the growing nuclei. Consequently, the homogeneous mixture surrounding the growing spherulites is depleted by the polymer (as shown by the arrow from the BC curve into the red region in Figure 5d). When the mixture depletes due to the polymer so that it crosses the binodal, liquid–liquid phase separation occurs in the regions outside the growing spherulites (Figure 5b). As a result, the droplets of the polymer-lean liquid (shown in Figure 5 as blue circles) appear in the polymer-rich liquid surrounding the spherulites. A similar phenomenon was found for the liquid–liquid phase separation induced by crystallization for the first time by Tanaka et al. [83] for the binary mixtures of poly-ε-caprolactone and polystyrene. Figure 5e shows a reprinted of the cited paper, showing an optical micrograph of the poly-ε-caprolactone spherulite with just-formed droplets of the polystyrene-rich phase on its surface. Finally, crystallization covers the entire volume of the sample due to both the continued growth of the already existing-spherulites (shown by dotted lines in Figure 5c) and the nucleation and growth of the new spherulites in the polymer-enriched dispersion phase of the emulsion (Figure 5c). An example of the formed structure is shown in Figure 5f (high-magnification SEM image of the bottom part of the sample 15P–0N–70S cross-section).

One more question requires attention before this section is concluded, pertaining to why the structure formation mechanism gradually changes from the scheme shown in Figure 4 to the scheme in Figure 5 as the DMAc concentration in the precipitation bath increases and as the distance from the surface layer increases. To answer this question, an analogy for the ternary-phase diagram and the temperature-composition phase diagram for the SC polymer–solvent mixture can be used.

**Figure 6:**
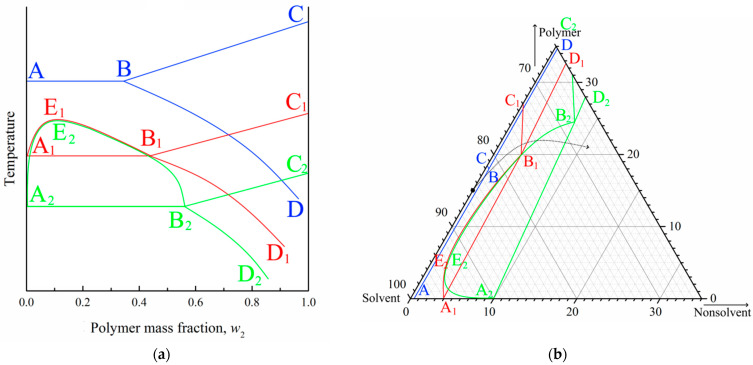
(**a**) Schematic phase diagrams of the binary SC polymer–solvent mixture obtained in conditions close to equilibrium (blue curves), at a certain low cooling rate (red curves), and at a high but finite cooling rate (green curves). (**b**) Fragments of experimental quasiequilibrium ternary phase diagram for the PVDF–DMAc–water mixture at room temperature (blue curves), and schematic dynamic phase diagrams of the same mixture at the same temperature under conditions of relatively slow mass transfer (red curves) and fast mass transfer (green curves).

It is well known that the position of the crystallization curve (ABC) on a phase diagram (Figure 6a) depends on the cooling rate. Specifically, an increase in the cooling rate results in a shift in the crystallization curve to a lower temperature (at first to the A_1_B_1_C_1_ and then to the A_2_B_2_C_2_). In other words, the supercooling degree required for the initiation of polymer crystallization increases with an increase in cooling rate. At the same time, the position liquid equilibrium binodal (A_1_E_1_B_1_; A_2_E_2_B_2_) is almost insensitive to the cooling rate [84,85]. Thus, the topology of the phase diagram transforms, as shown in Figure 6a. In this figure, the quasi-equilibrium phase diagram is blue, dynamic phase diagram at a certain cooling rate is red, and another dynamic phase diagram at higher cooling rate is green. 

In contrast to the binary phase diagrams, it is practically impossible to plot a dynamic ternary phase diagram. At the same time, for ternary mixtures at a fixed temperature, the mass transfer rate is an analogue to the cooling rate in binary mixtures, and supersaturation degree is an analogue of the supercooling degree. Thus, with an increase in the mass transfer rate, the topology of the ternary phase diagram should change, as shown in Figure 6b. In this figure, blue lines show a quasi-equilibrium phase diagram of the ternary mixture (Figure 1b), red lines show the hypothetic dynamic phase diagram at a low mass transfer rate, and green lines show the hypothetic dynamic phase diagram at a higher mass transfer rate.

Figure 6b also shows the so-called “composition path” of the mixture immersed in a precipitation bath (black arrow). This path consequently crosses the binodal (A_2_E_2_B_2_) and then crystallization line (A_2_B_2_) in the phase diagram for a high mass-transfer rate (green lines). For the phase diagram for a low mass-transfer rate (red lines), the composition path crosses the polymer crystallization line (B_1_C_1_) first, and then the swelling curve (B_1_D_1_).

In light of the above discussion, and taking into account that the mass transfer rate decreases with an increase in the DMAc concentration and increasing distance from the surface layer, the observed trends in structure transformation become quite expected.

#### 3.2.2. The Effect of Precipitation Bath Harshness on the Mechanical, Transport and Other Properties of the Membranes

The water contact angle of both surfaces of the membranes was found to be almost independent of the DMAc concentration in the precipitation bath. It was 70 ± 5° for both surfaces, which is typical of the PVDF membranes [86,87].

**Figure 7:**
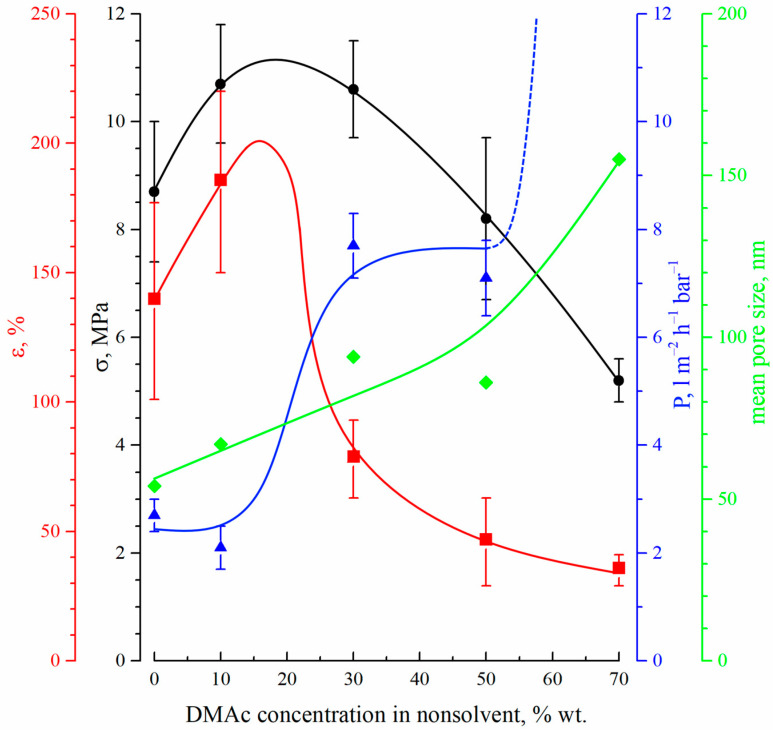
The dependencies of the tensile strength (black circles), elongation at break (red squares), permeance (blue triangles) and mean size of through pores (green diamonds) on the DMAc concentration in the precipitation bath.

Figure 7 shows the dependencies of the tensile strength (black circles), elongation at break (red squares), permeance (blue triangles) and mean size of through pores (green diamonds) on the DMAc concentration in the precipitation bath.

The mechanical properties (both tensile strength and relative elongation at break) of the membranes pass through a maximum with an increase in DMAc content in the precipitation bath. This fact can be explained by considering the morphology of the membranes. On the one hand, a decrease in the precipitation bath’s harshness results in a decrease in size and, after a certain point, even degeneration of the macrovoids (FLP) in the membrane structure. Since the macrovoids are large, defects in the membrane structure cause a decrease in their size and quantity, which leads to an increase in the mechanical properties. From the SEM images (Figure 2 and Figure 3), it can be seen that macrovoids are only present in samples 15P–0N–0S, 15P–0N–10S, and 15P–0N–30S. At the same time, a decrease in the precipitation bath’s harshness leads to the appearance of type 2 spherulites (Figure 5) and to an increase in their volume fraction in the matrix of the samples. It is well known that the structures formed due to polymer crystallization directly in the homogeneous solution (due to the so-called solid–liquid phase separation) are often characterized by a lower mechanical strength than those formed due to consecutive liquid–liquid phase separation and polymer crystallization [63,67]. Therefore, an increase in the type 2 spherulites’ volume fraction in the sample matrix leads to a decrease in the mechanical properties. A detailed analysis of the SEM images (Figure 2 and Figure 3) indicates that structures composed of type 2 spherulites are absent in samples 15P–0N–0S and 15P–0N–10S, start to appear in 15P–0N–30S, and occupy an increasingly large part of the structure in 15P–0N–50S and 15P–0N–70S. Thus, one of the trends (decrease in macrovoids’ quantity and volume) increases the mechanical properties, while the other (increase in type 2 spherulites’ volume fraction in the sample matrix) decreases the mechanical properties. A combination of these trends results in the appearance of the maximum dependencies of tensile strength and relative elongation at break in the DMAc concentration in the precipitation bath.

Figure 7 also shows that the mean size of through pores and permeance of the membranes increase with an increase in DMAc content in the precipitation bath. Moreover, the permeance of the sample 15P–0N–70S was higher than that of the others by almost two orders of magnitude (not shown in Figure 7). This high value is probably related to the appearance of macroscopic defects in the membrane structure. From the data on the morphology of the membranes, it can be concluded that the observed trends in the variation in transport properties are a function of the surface layer structure, where both porosity and pore size increase with an increase in DMAc content in the precipitation bath. It should be noted that the permeance of the prepared membranes (except for 15P–0N–70S) is several times (or, in some cases, even one or two orders of magnitude) lower than that of the cutting-edge PVDF membranes with a similar mean pore size (see, for example, [20,21,22,23,24]). However, additional chemical modification, additives in the dope solution, modifications of the casting process, etc., were necessary to achieve such permeance values. In our work, we purposefully omitted such approaches to enable a detailed thermodynamic investigation of specific parameters (in this case, the harshness of the precipitation bath). Despite the quite low permeance of the membranes prepared under these specific conditions, the revealed general trends of the variation in membrane properties with changes in the precipitation bath’s harshness may be useful for further studies focusing on the preparation of advanced membranes.

#### 3.2.3. Effect of Precipitation Bath Harshness on the Membranes’ Crystalline Structure

**Figure 8:**
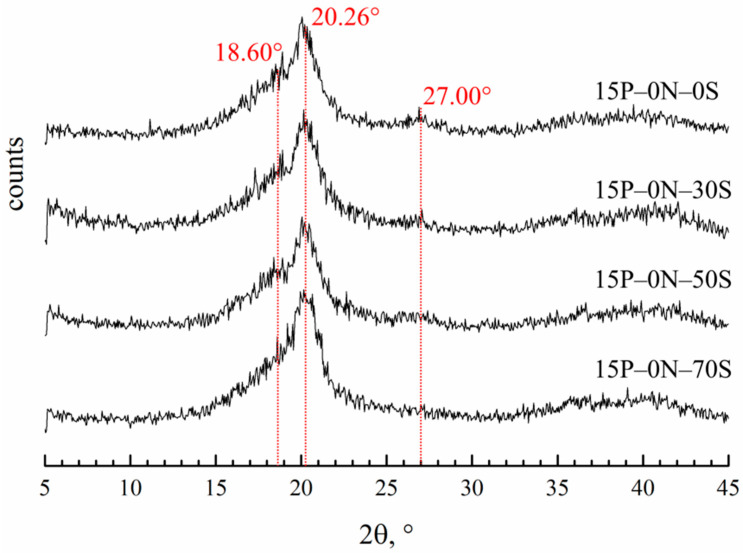
XRD patterns of the membrane samples prepared via immersion in precipitation baths of different compositions. Sample code is shown to the right of the curves.

Figure 8 shows the diffraction patterns of the membranes prepared by immersion in precipitation baths of different compositions.

Diffractograms of all the samples contain three main reflections. The reflection at 20.26° is the same for all three of the main crystalline modifications of PVDF (α, β and γ). Reflections at 18.6° and 27° are related to the crystalline structure of the α-modification. It is hard to judge the content of the β-modification, since the 110 reflection is the same as that of the β- and γ-modifications. However, the absence of a 001 reflection corresponding to the β-modification allows for us to conclude that there cannot be many β-modification crystallites in the samples. The ratios of the integral intensities of the crystalline reflections and amorphous halo were used to estimate the overall degree of crystallinity in the samples. From the theoretical ratios of the α-modification reflections, the intensity of the 110 reflection corresponding solely to the α-modification was calculated. The calculated intensity of this reflection was compared to the intensity of the real peak at 20.26° and the fraction of α-modification crystallites in the samples was estimated. In the Table 2, the data on crystallinity degree and the fraction of α-modification compared to all other modifications in the membrane samples are summarized.

One can see that the crystallinity degree passes through a weak maximum with an increase in DMAc content in the precipitation bath. This factor may further contribute to the observed trend of a variation in mechanical properties with decreases in the precipitation bath’s harshness. A fraction of α-crystallites also decreases in this series. This agrees well with the fact that the crystallization of PVDF from its mixtures with DMAc mainly yields γ-crystallites [69,70]. The higher the concentration of DMAc in the precipitation bath, the stronger its effect on the crystallization behavior of PVDF.

### 3.3. Effect of the Solvent Thermodynamic Affinity to the PVDF on the Membrane Morphology and Properties

The thermodynamic affinity of the solvent in relation to the polymer was controlled by the addition of different amounts of water to the DMAc. The amount of water that was added and the sample preparation technique are described in Section 2.2.1. It should be noted that all the mixtures, after cooling to 25 °C and immediately prior to the precursor formation, remained homogeneous and optically transparent. However, as follows from the phase diagram in Figure 1b, all the mixtures containing water were located in the green region of the phase diagram. Therefore, their homogeneous state at room temperature is not equilibrium. On more argument for this is that, even if stored in a closed vessel (so that water vapor cannot be sorbed by the mixture), all the mixtures containing water become turbid and gel-like during a time ranging from 1 h (for the mixture 15P–6N–0S) to two days (for the mixture 15P–2N–0S).

#### 3.3.1. Effect of Nonsolvent Concentration in the Dope Solution on the Morphology of the Membranes

**Figure 9:**
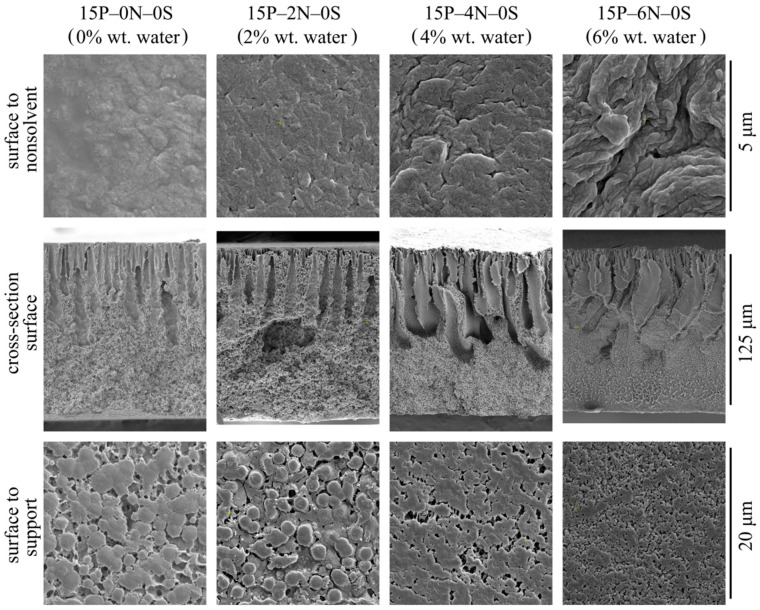
SEM images of the surface that was in contact with the nonsolvent (**upper row**), cross-section (**middle row**), the PET substrate (**bottom row**) of the membrane samples prepared from dope solutions with different water concentrations in the cosolvent. Sample code and water mass fraction in the cosolvent are shown above the images.

Figure 9 shows the SEM images of the surface that was in contact with the precipitation bath, the membrane cross-section, and the surface that was in contact with the PET substrate of the membrane samples 15P–0N–0S, 15P–2N–0S, 15P–4N–0S, and 15P–6N–0S.

First, an increase in water concentration in the dope solution leads to an increase in the upper surface’s porosity and roughness. The surface that was in contact with the PET substrate differs only in terms of the size of spherulites it is composed of. Specifically, the size of spherulites on the bottom surface decreases with the increase in water content in the dope solution. In the cross-section of all the membrane samples, FLP is observed to grow from the upper surface.

**Figure 10:**
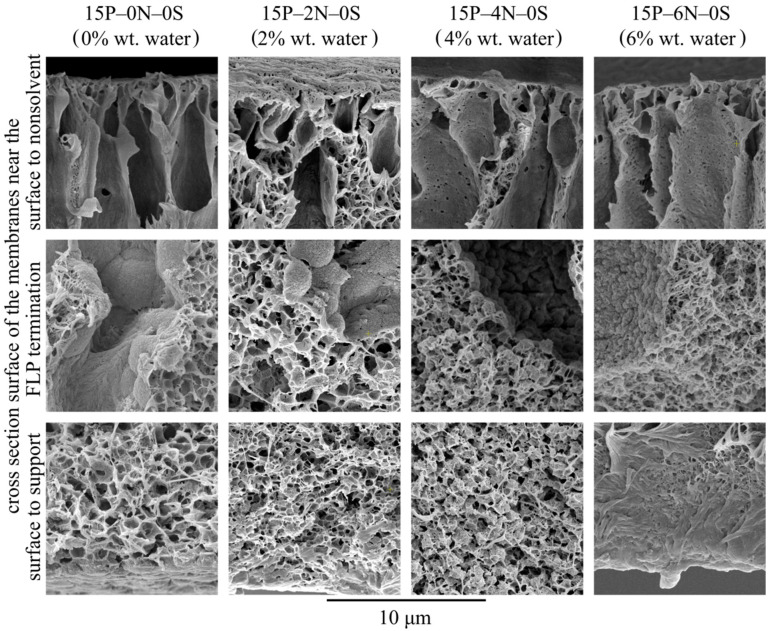
SEM images of the cross-section near the surface that was in contact with the precipitation bath (**upper row**), near the middle of the sample (**middle row**), and near the surface that was in contact with the PET substrate (**bottom row**) of the membranes prepared from dope solutions with different water concentrations in the cosolvent. Sample code and water mass fraction in the cosolvent are shown above the images.

Let us consider the morphology of the membranes in more detail using the higher-magnification SEM images of their cross-sections shown in Figure 10. In these micrographs, it is clear that all the samples have a very similar structure near the surface that was in contact with the precipitation bath. The mechanism of this structure’s formation is discussed and illustrated i Figure 4. Samples 15P–0N–0S and 15P–2N–0S are very similar except for the fact that the mean pore size of the sponge-like structure in the latter sample is a little lower. This could be due to the fact that an increase in the amount of nonsolvent in the dope solution leads to an increase in the nucleation rate at the early stages of liquid–liquid phase separation.

Although samples 15P–4N–0S and 15P–6N–0S also contain FLP, their structure is noticeably different from that discussed above. In sample 15P–4N–0S, the structure between and below the FLP is composed of a large amount of small spherulites, encircled by a sponge-like structure, while in 15P–6N–0S, similar spherulites were not detected.

From the thermodynamic point of view, the addition of solvent to the precipitation bath (discussed in Section 3.2) retards the mass transfer processes (and thus delays and slows down the phase separation), which results in the degeneration of finger-like pores and an increase in surface layer porosity. In contrast, the addition of water to the dope solution (described in this section) favors the phase separation process. This is because, for dope solutions already containing water, a lower amount of nonsolvent needs to penetrate into the membrane precursor to induce phase separation. Furthermore, as stated above, phase separation in a nonequilibrium solution may have started at the moment of film casting or upon its immersion into the precipitation bath. This may also contribute to the drastic difference observed in the cross-section structure of the 15P–4N–0S and 15P–6N–0S samples.

#### 3.3.2. Effect of the Nonsolvent Concentration of the Dope Solution on the Transport and Mechanical Properties of the Membranes

**Figure 11:**
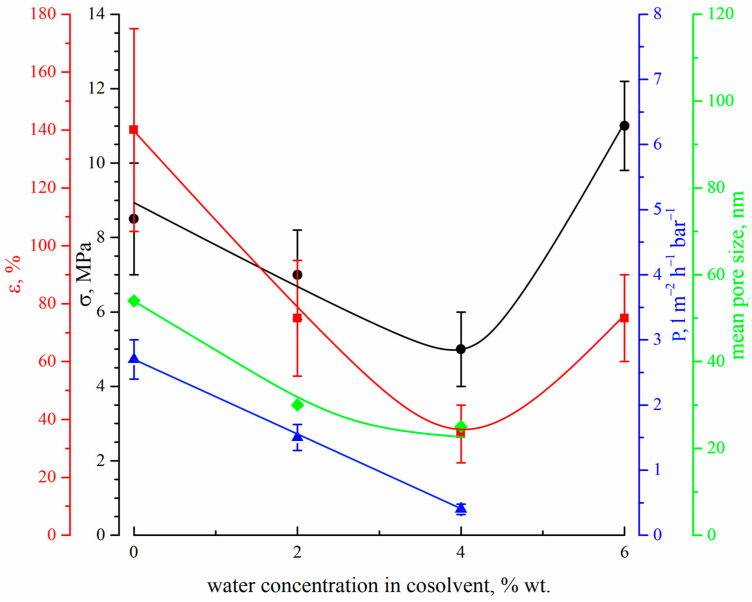
The dependencies of the tensile strength (black circles), elongation at break (red squares), permeance (blue triangles) and mean size of through pores (green diamonds) on the water concentration in the cosolvent used for the preparation of dope solution.

Figure 11 shows the dependencies of the tensile strength (black circles), elongation at break (red squares), permeance (blue triangles) and mean size of through pores (green diamonds) on the concentration of water in the cosolvent used for dope solution preparation.

Both tensile strength and elongation at break surpass the minimum (for the 15P–4N–0S sample) with an increase in water content in the dope solution. The observed trend can be explained by the transformations of the membrane morphology. The initial decrease in the mechanical properties is probably related to the gradual transformation of the structure between and below the FLP, from sponge-like (result of consecutive liquid–liquid phase separation and polymer crystallization) to the one containing type 2 spherulites (which is the result of polymer crystallization from a homogenous mixture with the solvent and nonsolvent). The latter structure is known to be less strong [63,67], and thus a decrease in mechanical properties is expected. The increase in the mechanical properties upon transition to the 15P–6N–0S sample can be explained by at least two factors. Firstly, the porous structure between and below FLP in this sample becomes sponge-like (i.e., stronger) again. Secondly, as can be seen from Figure 9, this sample experienced a great shrinkage during the drying stage, which led to a decrease in its overall porosity. The calculated shrinkage values for all samples, except 15P–4N–0S and 15P–6N–0S, were 30 ± 2%, while for these two samples, it was 41 ± 2% and 48 ± 2%, respectively. Considering that tensile strength is calculated as the relation between breaking force and the area of the sample cross-section (including the area occupied by pores), it is to be expected that this less porous sample would have higher strength and break elongation.

Figure 11 also shows that an increase in the nonsolvent concentration in the dope solution leads to a decrease in both through pore size and permeance. We were not able to measure the transport properties of sample 15P–6N–0S, which is to be expected when considering its low porosity (see cross-section of the bottom part of this sample in Figure 10). This is probably related to the trends in the change in mean pore size in the membranes. The permeance of the membranes prepared in this set of experiments was also quite low. It should be noted, however, that both mean pore size and permeance decreased when water was added to the dope solution, probably due to an increase in the shrinkage of the samples. Obviously, shrinkage could be reduced in several ways; for example, by annealing the wet membrane before drying, by changing the parameters of the drying process, and by replacing water with another liquid with lower surface tension. The membranes obtained in these ways would be characterized by their higher permeance but detailed research on these issues is beyond the scope of the present paper.

#### 3.3.3. Effect of the Nonsolvent Concentration in the Dope Solution on the Membranes Crystalline Structure

**Figure 12:**
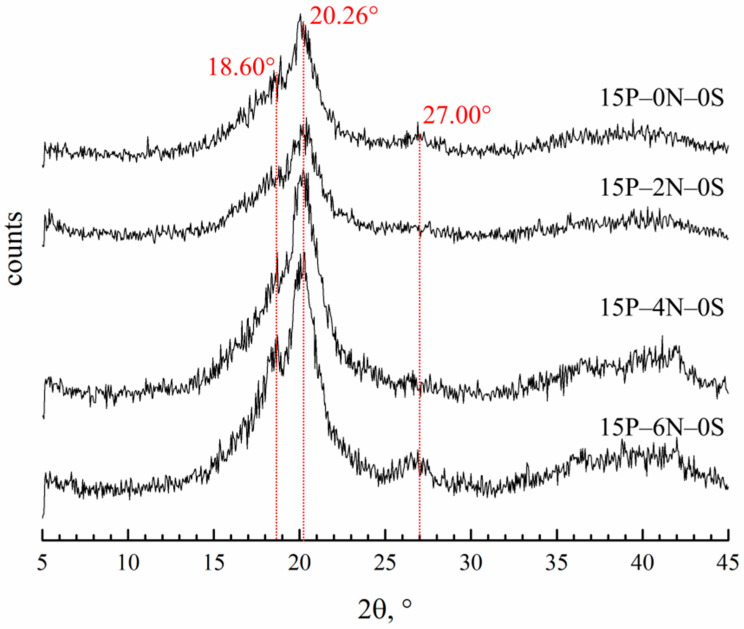
XRD patterns of the membrane samples prepared from dope solutions with different water concentrations in the cosolvent. The sample code is shown to the right of the curves.

Figure 12 shows the diffractograms of membrane samples prepared using the dope solutions containing various amounts of water. In Table 3, data on the degree of crystallinity and fraction of α-modification in the crystalline fraction of the membrane samples are presented. The crystalline structure of these samples contains α-, β- and γ-modifications of PVDF. Due to the superposition of reflections for the β- and γ-modifications, it is difficult to estimate their quantitive content. Nevertheless, it can be concluded that the crystalline structure of all samples mostly consists of γ-crystallites, while β-form crystallites do not make a significant contribution to the overall crystallinity of the samples.

A slight increase in the overall degree of crystallinity with an increase in water content in the dope solution could be a result of the recrystallization processes that occurred due to shrinkage during the drying stage of membrane formation. It also can partially explain the increased tensile strength of sample 15P–6N–0S.

## 4. Conclusions

In the present work, a systematic study of the precipitation bath’s harshness, as well as of the polymer–solvent’s thermodynamic affinity with the structure, transport, crystalline and physico-mechanical properties of the membranes, was performed. The PVDF membranes were prepared via NIPS using DMAc as a solvent and water as the nonsolvent. The harshness of the precipitation bath was varied by the addition of different amounts of solvent (DMAc) to the nonsolvent (water). Polymer–solvent affinity was controlled by mixing the solvent (DMAc) with the nonsolvent (water) to form a cosolvent with decreased affinity with the PVDF.

Based on the plotted at 25 °C ternary phase diagram for the PVDF–DMAc–water mixture, a new understanding of the mechanism of membrane structure formation is proposed.

It was shown that an increase in DMAc concentration in the precipitation bath leads to the transformation of membrane morphology from the asymmetric, which is standard for the ultrafiltration membranes and composed of a selective skin layer, a layer with FLP and a layer with a sponge-like structure to a more symmetric structure that does not contain FLP. It is established that the transcrystalline structure is formed in the surface layer of the membranes, while, in the bulk of the membrane, the spherulites with different morphologies can be formed depending on the parameters of the membrane preparation process. It is established that a decrease in the precipitation bath’s harshness leads to an increase in the membrane transport properties (mean pore size from ~60 to ~150 nm and permeance from ~2.8 to ~8 L m^−2^ h^−1^ bar^−1^) while the mechanical properties of the membranes pass through a maximum (tensile strength changes from ~9 to ~11 and to 6 MPa, while elongation at break increases from ~140 to ~190% before dropping to values as low as 35%).

An increase in water concentration in the cosolvent media causes a significant change in the membrane morphology, which remains asymmetric. At the same time, this results in a decrease in both transport (mean pore size changed from ~55 to ~25 nm and permeance decreased from ~2.8 to ~0.5 L m^−2^ h^−1^ bar^−1^) and the mechanical properties of the membranes (tensile strength decreased from ~9 to ~6 MPa and elongation at break decreased from ~140 to ~35%). It was shown that a slight increase in mechanical properties with the addition of 5.1% wt. water to the dope solutions was shown to be the result of increased sample shrinkage. An analysis of the trends obtained in the membrane properties’ variation allows for us to conclude that, for preparation of the high-quality membranes, pure DMAc as a solvent and a precipitation bath composed of 10–30% wt. of the solvent are preferable. XRD studies show that the crystallization of PVDF in all obtained membranes mainly occurs with the formation of γ-modification (approximately 70%) while the content of α-modification is sensitive to the parameters of the membrane formation process.

As a result of the investigations, the general principles and thermodynamic interpretation of the structure’s formation mechanism were formulated, which may be useful for further studies involving variations in the preparation process parameters to increase the membrane permeance.

## Figures and Tables

**Table 1:** Compositions of the dope solutions and precipitation baths used.

Sample Code	PVDF Mass Fraction in Dope Solution, wt. %	DMAc Mass Fraction in Dope Solution, wt. %	Water Mass Fraction in Dope Solution, wt.%	DMAc Mass Fraction in Precipitation Bath, wt. %	Water Mass Fraction in Precipitation Bath, wt. %
15P–0N–0S	15	85	0	0	100
15P–0N–10S	15	85	0	10	90
15P–0N–30S	15	85	0	30	70
15P–0N–50S	15	85	0	50	50
15P–0N–70S	15	85	0	70	30
15P–2N–0S	15	83.3	1.7	0	100
15P–4N–0S	15	81.6	3.4	0	100
15P–6N–0S	15	79.9	5.1	0	100

**Table 2:** Crystallinity degree and the fraction of α-crystallites in the crystalline part of the samples.

Sample Code	Crystallinity Degree, %	Fraction of α-Modification Crystallites, %
15P–0N–0S	50	20
15P–0N–30S	55	15
15P–0N–50S	56	15
15P–0N–70S	50	7

**Table 3:** Crystallinity degree and the fraction of α-crystallites in the crystalline part of the samples.

Sample Code	Crystallinity Degree, %	Fraction of α-Modification Crystallites, %
15P–0N–0S	50	20
15P–2N–0S	53	15
15P–4N–0S	53	10
15P–6N–0S	55	20

## Data Availability

The data presented in this study are available on request from the corresponding author.
